# Socially Prescribed Perfectionism, Resilience, and Internet Gaming Disorder in Adolescents: 3-Wave Longitudinal Study

**DOI:** 10.2196/93412

**Published:** 2026-04-30

**Authors:** Peng Zheng, Zi Tao, Tao Yao, Luoxiang Fang, Min Zhao

**Affiliations:** 1 Second Affiliated Hospital & Yuying Children's Hospital of Wenzhou Medical University, No. 109 Xueyuan West Road, Wenzhou, Zhejiang Province, 325000, China, 86 15057765818

**Keywords:** socially prescribed perfectionism, internet gaming disorder, resilience, sex differences, adolescents

## Abstract

**Background:**

Internet gaming disorder (IGD) is increasingly prevalent among adolescents. Although socially prescribed perfectionism (SPP) and resilience are both related to IGD, longitudinal evidence on their temporal relationships and underlying mechanisms remains limited.

**Objective:**

This study aimed to examine the longitudinal associations among SPP, resilience, and IGD in Chinese adolescents; test the mediating role of resilience; and explore potential sex differences.

**Methods:**

A 3-wave prospective longitudinal study was conducted among students from 4 middle schools in Zhejiang Province, China. Adolescents who had played online games in the past 12 months were recruited using convenience sampling. Data were collected at 6-month intervals: time 1 (T1; March 2024), time 2 (T2; September 2024), and time 3 (T3; March 2025). A total of 1332 Chinese adolescents (875/1332, 65.7% male; mean age 13.61, SD 0.70 years) participated in the baseline survey. SPP, resilience, and IGD were assessed using the Hewitt-Flett Multidimensional Perfectionism Scale-Short Form, the 10-item Connor-Davidson Resilience Scale, and the 9-item *Diagnostic and Statistical Manual of Mental Disorders* (Fifth Edition) IGD Checklist, respectively. Cross-lagged panel model (CLPM) and multigroup analyses across sex were conducted.

**Results:**

All statistical tests were 2-tailed with *α*=.05. The CLPM demonstrated good fit to the data (χ²_38_=163.34; comparative fit index [CFI]=0.945; Tucker-Lewis index [TLI]=0.932; root mean square error of approximation [RMSEA]=0.054; standardized root mean square residual [SRMR]=0.047). Higher SPP predicted later IGD (T1→T2: *β*=0.10, 95% CI 0.04 to 0.16, *P*<.001; T2→T3: *β*=0.09, 95% CI 0.03 to 0.15, *P*=.004) and lower resilience (T1→T2: *β*=−0.09, 95% CI −0.16 to −0.02, *P*=.007; T2→T3: *β*=−0.12, 95% CI −0.18 to −0.06, *P*<.001). In contrast, SPP was not significantly predicted by prior IGD nor resilience. Higher resilience predicted lower subsequent IGD (T1→T2: β=−0.09, 95% CI −0.15 to −0.03, *P*=.001; T2→T3: *β*=−0.09, 95% CI −0.15 to −0.03, *P*=.001), whereas higher IGD predicted lower subsequent resilience (T1→T2: *β*=−0.19, 95% CI −0.27 to −0.11, *P*<.001; T2→T3: *β*=−0.09, 95% CI −0.15 to −0.03, *P*=.003). Bootstrapped mediation analysis showed a significant indirect effect of SPP at T1 on IGD at T3 via resilience at T2 (*β*=0.008, 95% CI 0.004 to 0.012, *P*=.005). However, multigroup analyses revealed no significant sex differences.

**Conclusions:**

This study provides novel insights into the longitudinal associations among SPP, resilience, and IGD in adolescents. Unlike previous research based mainly on cross-sectional data, this 3-wave CLPM study clarifies the temporal relationships among these variables and shows that resilience mediates the association between SPP and subsequent IGD. These findings advance the field by identifying a temporal psychological pathway underlying adolescent IGD. They also have practical implications for early screening and for developing resilience-focused interventions for adolescents at risk of IGD.

## Introduction

### Background

With the rapid expansion of digital entertainment, online gaming has become one of the most prevalent leisure activities among adolescents [1]. For a subset of individuals, however, excessive and poorly controlled gaming may develop into a maladaptive behavioral pattern with significant functional impairment [2]. Internet gaming disorder (IGD) refers to a persistent and recurrent pattern of gaming behavior marked by impaired control, prioritization of gaming over other activities, and continuation of play despite negative consequences [3]. Recent epidemiological evidence indicates that IGD has become an increasingly salient public health concern among adolescents. A recent meta-analysis indicated that the pooled prevalence of IGD is about 10% among Chinese adolescents [4]. Empirical research further indicates that IGD is associated with a range of negative outcomes, including impaired academic performance, sleep disturbances, depression, and anxiety [5-7]. These findings highlight the importance of identifying the antecedents of IGD to inform early prevention and intervention efforts.

### Socially Prescribed Perfectionism and IGD

Perfectionism is widely recognized as a multidimensional construct [8]. According to the Hewitt-Flett model, it comprises self-oriented perfectionism, which involves imposing exceptionally high standards on oneself; other-oriented perfectionism, which refers to placing unrealistic standards on others; and socially prescribed perfectionism (SPP), which reflects the belief that others expect one to be perfect and that acceptance depends on meeting these external standards [9-11]. Although all 3 dimensions may be relevant to adolescent development, this study focused specifically on SPP. Among these dimensions, SPP is most directly linked to external pressure, perceived social demands, and evaluative concerns, making it particularly salient during adolescence, a developmental period marked by heightened sensitivity to interpersonal evaluation and social approval [10]. In this study, SPP was conceptualized as a socially driven cognitive-personality vulnerability factor that reflects adolescents’ perceived external expectations and evaluative pressure [12]. Prior research has consistently identified SPP as the most maladaptive dimension of perfectionism, showing robust associations with poorer psychological well-being, greater psychological distress, and behavioral maladjustment [13,14]. In addition, SPP among adolescents and young adults has increased substantially over time, highlighting its growing developmental significance [15].

The focus on SPP is also theoretically relevant for understanding adolescents’ vulnerability to IGD. According to the Perfectionism Social Disconnection Model, individuals with high levels of SPP tend to perceive intense external demands and fear harsh evaluation from others [12]. To avoid criticism and preserve a flawless image, they may engage in self-concealment and emotional suppression [16], which can undermine authentic interpersonal relationships and contribute to persistent feelings of social disconnection [12]. These interpersonal and emotional difficulties may, in turn, increase adolescents’ reliance on online environments as a means of coping [17]. From the perspective of compensatory internet use theory [18], adolescents may turn to online gaming to escape real-life stress, regulate negative emotions, and compensate for unmet social and psychological needs. For adolescents with high levels of SPP, online games may provide a relatively controllable context in which evaluation can be avoided or managed more easily while also offering temporary experiences of competence, achievement, and social connection [19]. Over time, this pattern of using gaming to cope with distress and interpersonal insecurity may increase vulnerability to problematic gaming behaviors and IGD [20]. Therefore, SPP may be understood as a socially driven vulnerability factor that contributes to the development of IGD in adolescents.

Empirical evidence supports the association between perfectionistic tendencies and problematic online behaviors among adolescents and university students. Studies have shown that maladaptive perfectionism is positively associated with IGD symptoms and higher levels of internet-related behavioral problems [21,22]. Research also indicates that individuals with stronger perfectionistic concerns rely more on online activities to regulate negative affect [23]. Additionally, a 12-month follow-up study involving 465 highly engaged Australian gamers found that higher baseline levels of perfectionistic gaming-related cognitions significantly predicted subsequent increases in problematic gaming behaviors [24]. Despite these observations, most studies examining perfectionism and IGD remain cross-sectional, limiting the ability to determine temporal direction or causal mechanisms. Besides, few studies have directly examined the association between SPP and IGD. Therefore, it is necessary to use longitudinal methods to clarify such association among adolescents.

### Resilience as a Potential Mediator

Another notable gap in the literature is the insufficient research exploring the psychological mechanisms linking SPP to IGD. Although previous studies have identified SPP as a risk factor for various maladaptive behavioral outcomes, including problematic internet and gaming behaviors, the mediating pathway that explains this association remains underexamined. One potential mediator is resilience. Resilience is generally defined as a positive psychological trait that enables individuals to adapt successfully to stress, adversity, or failure through effective coping strategies and emotional regulation and may play a key role in this relationship [25,26]. Rather than representing a fixed personal attribute, resilience reflects a dynamic capacity to recover from setbacks, maintain psychological stability, and mobilize internal and external resources when confronted with perfectionism-related stress [27].

The model of compensatory internet use [18] provides a useful theoretical framework for understanding why resilience may mediate the association between SPP and IGD. It proposes that individuals may engage in online activities not only for enjoyment but also to compensate for offline stress, negative emotions, and unmet psychological needs [18,28]. In other words, problematic online behaviors may emerge when the internet is used as a coping tool to manage distress that individuals feel unable to handle effectively in real life [18]. From this perspective, resilience is highly relevant because it reflects the capacity to cope adaptively with stress and emotional challenges [29]. Adolescents with higher resilience are generally better able to regulate negative emotions, recover from setbacks, and rely on effective coping strategies [30], which may reduce the need to use online gaming as an escape or emotional outlet [31]. In contrast, adolescents with lower resilience may be less able to manage perfectionism-related stress in adaptive ways and therefore more likely to turn to online gaming as a compensatory strategy to avoid pressure, relieve distress, or obtain temporary feelings of competence and control [20]. This process may be particularly relevant for adolescents with high levels of SPP and who tend to perceive elevated external expectations, fear negative evaluation, and experience greater emotional vulnerability in academic, social, and performance contexts [32]. Over time, repeated reliance on gaming for emotional relief may reinforce maladaptive coping patterns and increase the risk of IGD, especially given the immersive and performance-based features of online games, which provide immediate feedback, achievement, and social affirmation [20].

This interpretation is also consistent with the stress-vulnerability-protective factors model, which emphasizes that psychological outcomes are shaped by the interaction between stressors, vulnerability factors, and protective resources [33]. Within this framework, SPP may be understood as a stress-related vulnerability factor [12], whereas resilience may function as a protective resource that helps adolescents maintain psychological adjustment in the face of external pressure and evaluative stress [30]. Accordingly, adolescents with high levels of SPP may be at greater risk of IGD partly because perfectionism-related stress undermines resilience, thereby increasing reliance on maladaptive coping behaviors such as online gaming.

Existing empirical findings provide support for these theoretical accounts. Prior research has shown that higher levels of SPP are associated with lower resilience, as individuals who fear external evaluation or criticism tend to exhibit heightened stress sensitivity and reduced adaptive coping skills [34]. Resilience, in turn, has been found to buffer against addictive or compulsive technology use. Several studies have demonstrated that lower resilience predicts greater vulnerability to problematic gaming and internet behaviors among adolescents and young adults [31,35]. A large-scale study found that stress originating from adverse childhood experiences increased the risk of IGD by undermining resilience, supporting a stress-resilience pathway to IGD [36]. Complementary findings suggest that resilience and stress jointly influence IGD tendencies [37], highlighting the importance of coping resources in shaping susceptibility to gaming-related problems. Despite these advances, important gaps remain. Most existing studies have relied on cross-sectional designs, which capture only static associations and cannot establish temporal precedence among SPP, resilience, and IGD. In addition, few studies have integrated the compensatory internet use framework with longitudinal tests of resilience as a mediator between SPP and IGD.

### Sex Differences

Researchers have highlighted that sex plays a significant role in shaping vulnerability to problematic gaming. Specifically, males tend to report higher levels of IGD than females [38], show greater preference for performance-oriented gaming activities, and exhibit stronger competitiveness in digital environments [39]. These sex differences may reflect distinct psychological needs that drive gaming engagement. Females are more likely to engage in digital activities for interpersonal connection and emotional satisfaction, whereas males tend to use gaming to pursue competition, achievement, and self-enhancement motives [40]. Moreover, researchers have suggested that the situational factors contributing to addictive gaming may vary by sex. For instance, SPP has been shown to predict problematic online behaviors through heightened concern with external evaluation, and this effect appears particularly salient among males [41]. Similarly, the protective effects of psychological resilience against problematic internet use or problematic gaming have been found to be stronger among females than males [41]. However, most of these studies have been cross-sectional, limiting our understanding of whether these sex-related mechanisms persist over time. Although sex differences in general problematic internet behaviors have been widely examined, very few studies have specifically investigated whether these differences extend to the longitudinal associations between SPP, resilience, and IGD. Given that IGD strongly emphasizes achievement-oriented rewards and performance feedback, it is important to examine whether similar sex-specific patterns exist in these pathways.

### This Study

Given this background, this study used a cross-lagged panel model (CLPM) with a 3-wave longitudinal design to examine the longitudinal association between SPP and IGD and further test whether resilience mediates this relationship. It also explored potential sex differences in the associations among SPP, resilience, and IGD in adolescents. The following hypotheses were proposed: (H1) Higher levels of SPP positively predict subsequent IGD across adjacent waves; (H2) resilience mediates the longitudinal relationship between SPP and IGD; and (H3) the associations among SPP, resilience, and IGD differ by sex.

## Methods

### Study Design, Participants, and Setting

This study was designed as a 1-year prospective cohort study and conducted among students from 4 middle schools in Zhejiang Province, China. The recruitment setting was school-based, and participants were recruited from regular classroom populations in these 4 schools. The schools were selected based on accessibility and willingness to participate in the study. Data were collected at three 6-month intervals: time 1 (T1; March 2024), time 2 (T2; September 2024), and time 3 (T3; March 2025). The 6-month interval between waves was informed by prior adolescent longitudinal studies using similar follow-up periods [42-44] and was considered appropriate for capturing meaningful short-term changes in psychological and behavioral variables in a school-based design. Participants were recruited through convenience sampling, with all eligible students in the selected schools invited to participate during regular class hours through coordination with teachers and school administrators. Trained field researchers administered the surveys during regular class hours using self-report questionnaires. Prior to survey administration, both students and their parents (or legal guardians) were provided with comprehensive information about the study’s aims, procedures, and confidentiality safeguards. Written informed consent was obtained from parents or guardians, while students were informed that submitting the completed questionnaire would be interpreted as assent. It was emphasized that participation was voluntary and that nonparticipation or withdrawal would have no consequences on their academic standing or school records.

**Figure 1.**
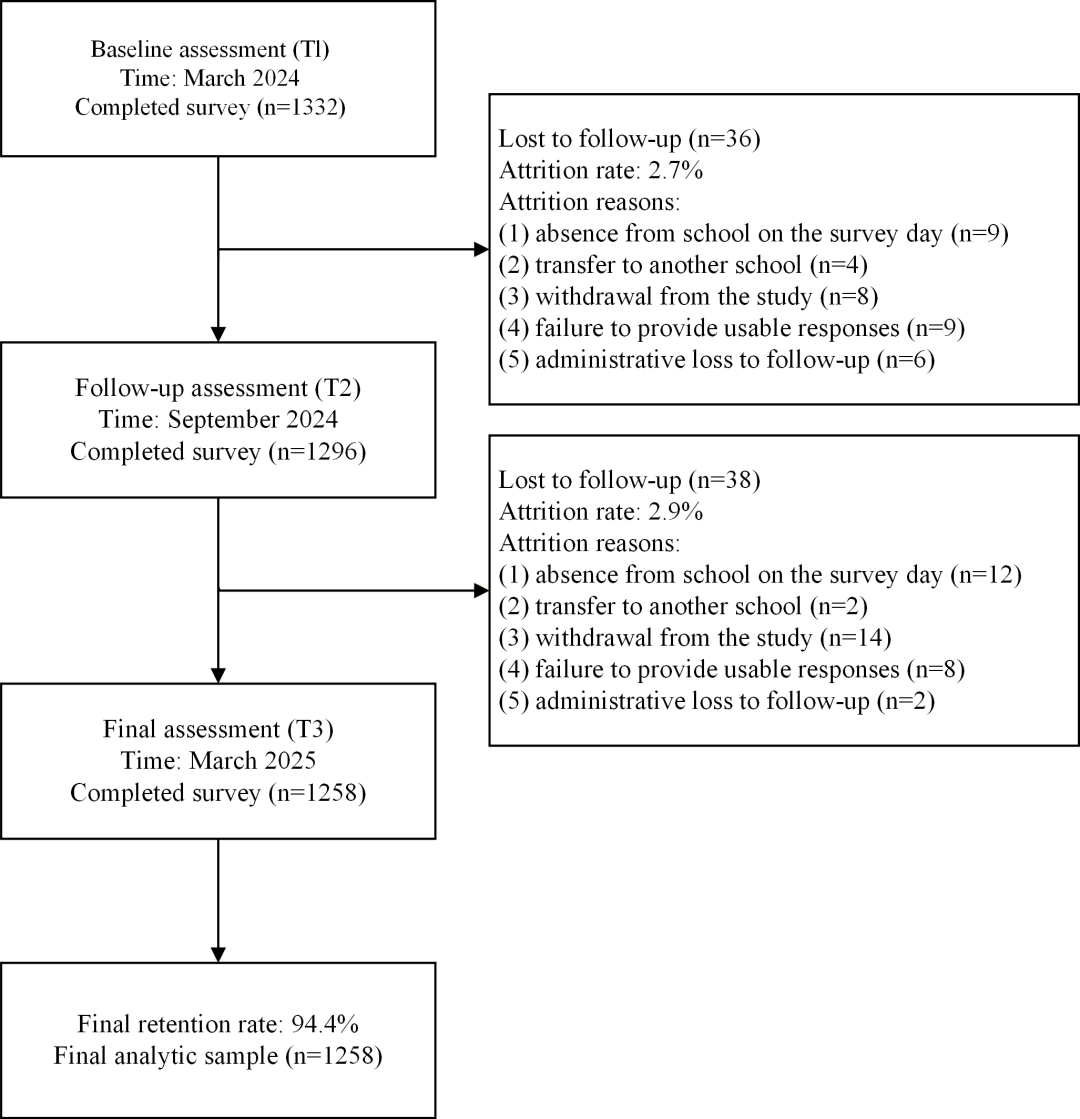
The participant flowchart.

At baseline (T1), 1332 adolescents (875/1332, 65.7% males; mean age 13.61, SD 0.70 years) completed the survey. At T2, 1296 participants remained in the study, indicating that 36 participants were lost between T1 and T2. The reasons for attrition from T1 to T2 were as follows: absence from school on the survey day (n=9), transfer to another school (n=4), withdrawal from the study (n=8), failure to provide usable responses (n=9), and administrative loss to follow-up (n=6). By T3, 1258 participants had completed all 3 assessments, indicating that a further 38 participants were lost between T2 and T3. The reasons for attrition from T2 to T3 were as follows: absence from school on the survey day (n=12), transfer to another school (n=2), withdrawal from the study (n=14), failure to provide usable responses (n=8), and administrative loss to follow-up (n=2). Overall, 74 participants were lost across the 3 waves. The participant flowchart is presented in Figure 1. Participants who provided valid data at least once were retained in the analyses to maximize statistical power and preserve sample representativeness. Missing data patterns and the rationale for the handling of missingness are reported in the Results section.

### Ethical Considerations

This study was approved by the Ethics Committee of the Second Affiliated Hospital and Yuying Children’s Hospital of Wenzhou Medical University (approval number: XMSQ-2024‐347). Before data collection, both students and their parents or legal guardians were provided with detailed information about the study aims, procedures, voluntary nature of participation, and confidentiality protections. Written informed consent was obtained from parents or legal guardians, and students were informed that submission of the completed questionnaire would be considered assent to participate. To protect privacy and confidentiality, student ID numbers were collected only for the purpose of matching responses across the 3 survey waves; these identifiers were stored securely and removed from the analytic dataset before analysis to ensure de-identification. Participation was voluntary, and participants could decline or withdraw without any academic consequences. No financial nor material compensation was provided for participation. In addition, no images in the manuscript nor supplementary materials contain identifiable information about individual participants.

### Studied Variables and Measurements

#### Demographic Variables

Demographic variables included age, sex, parental educational attainment, self-reported household income level, academic performance, single-parent family status, and average gaming time per month.

#### Internet Gaming Disorder

IGD was measured using the 9-item *Diagnostic and Statistical Manual of Mental Disorders* (Fifth Edition; *DSM-5*) IGD Checklist [45], which assesses gaming-related symptoms occurring within the past 12 months using a binary (yes/no) response format. The instrument evaluates core diagnostic features, including excessive preoccupation with gaming, withdrawal symptoms, tolerance, impaired control, prioritization of gaming over other activities, continued gaming despite adverse consequences, misrepresentation of gaming involvement, use of gaming as an escape from negative moods, and functional impairment resulting from gaming behavior. The checklist has been validated in adolescent populations and has demonstrated adequate reliability and validity [46]. For analysis, the 9 items were summed to create an observed total score reflecting IGD symptom severity, with higher scores indicating greater severity. In this study, the Cronbach α of this scale was 0.70 at T1, 0.75 at T2, and 0.81 at T3.

#### Resilience

Resilience was measured using the 10-item short form of the Connor-Davidson Resilience Scale, which assesses individuals’ capacity to adapt to stressors such as life changes, personal challenges, illness, pressure, failure, and emotional distress [47]. The Chinese version of the scale has demonstrated strong psychometric reliability and validity in prior research [48,49]. Items are rated on a 5-point Likert scale ranging from 0 (“not true at all”) to 4 (“true nearly all of the time”), yielding total scores between 0 and 40, with higher scores indicating greater resilience. In this study, the Cronbach α of this scale was 0.82 at T1, 0.85 at T2, and 0.90 at T3.

#### Socially Prescribed Perfectionism

SPP was assessed using the corresponding 5-item subscale of the Hewitt-Flett Multidimensional Perfectionism Scale Short Form [50]. Participants rated each item on a 7-point Likert scale ranging from 1 (“strongly disagree”) to 7 (“strongly agree”), with higher total scores indicating greater levels of SPP. The Chinese version of this subscale has demonstrated good reliability and validity in previous studies [51]. In this study, the Cronbach α of this scale was 0.83 at T1, 0.86 at T2, and 0.88 at T3.

### Preliminary Data Analyses

Attrition analyses were performed using *χ*^2^ tests for categorical variables and independent-samples *t* tests for continuous variables. Pearson correlation coefficients were calculated to examine the relationships among the main study variables. In the descriptive and correlational analyses conducted in SPSS (IBM Corp), listwise deletion was applied to address missing data. Given that the proportion of missing data was minimal (approximately 1%), this approach was considered suitable and unlikely to bias the results or substantially reduce the effective sample size.

### Measurement Invariance

Longitudinal measurement invariance was examined for SPP, resilience, and IGD. An acceptable configural invariance model was determined based on standard model fit criteria, including a comparative fit index (CFI) ≥0.90 and root mean square error of approximation (RMSEA) and standardized root mean square residual (SRMR) values ≤0.08 [52]. Metric and scalar invariance were supported when changes in model fit indices remained within recommended thresholds (ΔCFI≤0.01; ΔRMSEA≤0.015), indicating stability of factor loadings and item intercepts across time points and sex groups. Because scalar invariance was supported, the constructs were considered comparable across waves and were retained for the subsequent longitudinal structural analyses.

### CLPM and Mediation Analysis

In the longitudinal models, IGD was treated as an observed continuous summed score representing symptom severity rather than a categorical diagnostic variable. To investigate the longitudinal relationships among SPP, resilience, and IGD, an a priori CLPM was specified, which is well suited for testing directional and mediational effects in longitudinal datasets [52,53]. The model included autoregressive paths for SPP, resilience, and IGD across adjacent waves; cross-lagged paths among the 3 constructs across adjacent waves; and within-wave correlations among SPP, resilience, and IGD at each time point. Indirect effects were evaluated using bootstrapping procedures with 5000 resamples to generate 95% bias-corrected confidence intervals. Model adequacy was assessed using multiple goodness-of-fit indices, including *χ*²(*df*)≤5, CFI and Tucker-Lewis index (TLI)≥0.90, and RMSEA and SRMR≤0.08, consistent with established structural equation modeling guidelines [54].

### Multigroup Analyses

Multigroup analyses were conducted to examine sex differences in the longitudinal associations among SPP, resilience, and IGD. A series of nested models, each constraining a specific pathway, were compared with an unconstrained model in which all parameters were freely estimated. Sex differences were evaluated by comparing the fit of the unconstrained and constrained models. Following commonly recommended criteria for measurement and structural invariance testing, ΔCFI and ΔRMSEA were used as the primary indicators of model differences, with ΔCFI>0.01 and ΔRMSEA>0.015 indicating meaningful deterioration in model fit and, therefore, significant sex differences in the examined associations.

### Software, Estimation, and Missing Data Handling

Preliminary analyses were performed using SPSS version 26.0, and all longitudinal structural analyses were performed using Mplus 8.3. Before model estimation, the distributions of SPP, resilience, and IGD were examined across the 3 waves. Because no severe departures from normality were identified, maximum likelihood estimation was considered appropriate for the longitudinal analyses. For the longitudinal models estimated in Mplus, full information maximum likelihood (FIML) analysis was used to address missing data by retaining all available observations in model estimation [55]. No post hoc model respecification was undertaken for the primary longitudinal model. Statistical significance was set at a 2-tailed *P* value <.05.

### Sensitivity Analysis

In addition, multiple imputation was performed as a sensitivity analysis to examine the robustness of the findings to the handling of missing data. This approach has been widely used in longitudinal studies [56-58]. The results obtained from the imputed datasets were highly similar to those from the primary FIML analyses, indicating that the main findings were robust.

## Results

### Missing Data

At T1, the sample included 1332 adolescents; missing data were observed for age (11/1332, 0.8%), sex (4/1332, 0.3%), father’s educational level (11/1332, 0.8%), mother’s educational level (8/1332, 0.6%), academic performance (11/1332, 0.8%), SPP (7/1332, 0.5%), resilience (10/1332, 0.8%), and IGD (14/1332, 1.1%). The following variables did not have any missing data: self-reported household income, single-parent family status, and gaming time per month. At T2, the sample included 1296 adolescents.

Missing data at T2 were as follows: age, 9/1296, 0.7%; sex, 6/1296, 0.5%; father’s educational level, 12/1296, 0.9%; mother’s educational level, 9/1296, 0.7%; self-reported household income, 15/1296, 1.2%; academic performance, 7/1296, 0.5%; single-parent family status, 14/1296, 1.1%; gaming time per month, 8/1296, 0.6%; SPP, 14/1296, 1.1%; resilience, 8/1296, 0.6%; and IGD, 9/1296, 0.7%.

At T3, the sample included 1258 adolescents. Missing data at T3 were as follows: age, 6/1258, 0.5%; sex, 4/1258, 0.3%; father’s educational level, 7/1258, 0.6%; mother’s educational level, 7/1258, 0.6%; self-reported household income, 10/1258, 0.8%; academic performance, 6/1258, 0.5%; single-parent family status, 11/1258, 0.9%; gaming time per month, 6/1258, 0.5%; SPP, 10/1258, 0.8%; resilience, 12/1258, 1%; and IGD, 9/1258, 0.7%. The Little test for missing completely at random was nonsignificant (*χ*²_18_=15.55, *P*=.62), indicating that the missing data pattern was consistent with the missing completely at random assumption. Therefore, FIML was considered appropriate and was used in the longitudinal analyses to retain all available data.

### Distributions

Before fitting the CLPM, the distributions of the main study variables were examined across the 3 waves. The skewness and kurtosis values for SPP, resilience, and IGD indicated no severe departures from normality. Therefore, the use of maximum likelihood estimation in the subsequent longitudinal analyses was considered appropriate.

### Attrition Analyses

As shown in Table 1, attrition analyses comparing participants who remained in the study with those lost to follow-up revealed no statistically significant differences in age, sex, parental educational level, self-reported household income, academic performance, single-parent family status, gaming time per month, SPP, resilience, and IGD at baseline (all *P*>.05). These findings suggest that attrition was unlikely to substantially influence the subsequent longitudinal analyses.

**Table 1.** Attrition analyses comparing adolescents who completed all 3 waves of the longitudinal study and those lost to follow-up in a study of socially prescribed perfectionism (SPP), resilience, and internet gaming disorder (IGD).

Variable	Follow-up (n=1258)		Lost to follow-up (n=74)		*P* value^a^
Sex, n (%)					.92
Male	826 (65.8)		49 (67.1)		
Female	429 (34.2)		24 (32.9)		
Father’s educational level, n (%)					.83
Middle school or less	819 (65.7)		46 (62.2)		
Senior high school	338 (27.1)		22 (29.7)		
College or higher	90 (7.2)		6 (8.1)		
Mother’s educational level, n (%)					.19
Middle school or less	835 (66.8)		57 (77)		
Senior high school	309 (24.7)		13 (17.6)		
College or higher	106 (8.5)		4 (5.4)		
Self-reported household income level, n (%)					.11
Below average	260 (20.7)		11 (14.9)		
Average	711 (56.5)		51 (68.9)		
Above average	287 (22.8)		12 (16.2)		
Self-reported academic performance (percentile), n (%)					.09
0-20th	169 (13.6)		6 (8.1)		
21st-40th	271 (21.7)		11 (14.9)		
41st-60th (average)	324 (26)		17 (23)		
61st-80th	280 (22.5)		21 (28.4)		
81st-100th	203 (16.3)		19 (25.7)		
Single-parent family status, n (%)					.64
No	961 (76.4)		53 (71.6)		
Yes	137 (10.9)		10 (13.5)		
Not reported	160 (12.7)		11 (14.9)		
Gaming time per month (hours), n (%)					.78
<4	557 (44.3)		35 (47.3)		
4‐8	352 (28)		20 (27)		
8‐12	170 (13.5)		8 (10.8)		
12‐16	152 (12.1)		8 (10.8)		
>16	27 (2.1)		3 (4.5)		
Age (years), mean (SD)	13.61 (0.69)		13.67 (0.74)		.49
SPP, mean (SD)	16.83 (4.39)		17.07 (4.39)		.65
Resilience, mean (SD)	25.65 (9.90)		26.85 (8.45)		.32
IGD, mean (SD)	1.61 (1.78)		1.85 (1.87)		.24

a Based on available cases for each variable; missing data were excluded from the corresponding analysis.

### Descriptive Statistics of Main Variables

Across the 3 waves, mean values at T1, T2, and T3 were 16.83 (SD 4.39), 16.76 (SD 4.37), and 16.93 (SD 4.43), respectively, for SPP; 25.67 (SD 9.91), 25.55 (SD 9.61), and 25.59 (SD 10.18), respectively, for resilience; and 1.60 (SD 1.78), 1.52 (SD 1.86), and 1.66 (SD 2.12), respectively, for IGD.

### Correlation Analyses

As shown in Table 2, SPP was significantly and positively correlated with IGD across the 3 waves (*r*=0.24 to 0.35, all *P*<.001). Resilience was significantly and negatively correlated with SPP and IGD across 3 waves (*r*=–0.28 to –0.11, all *P*<.001).

**Table 2.** Pearson correlation analyses among socially prescribed perfectionism (SPP), resilience, and internet gaming disorder (IGD) across the 3 waves of the longitudinal study among Chinese adolescents.

	SPP at T1	SPP at T2	SPP at T3	Resilience at T1	Resilience at T2	Resilience at T3	IGD at T1	IGD at T2	IGD at T3
SPP at T1									
*r*	1	0.57	0.52	–0.28	–0.24	–0.17	0.30	0.27	0.24
*P* value	—^a^	<.001	<.001	<.001	<.001	<.001	<.001	<.001	<.001
SPP at T2									
*r*	0.57	1	0.55	–0.21	–0.24	–0.20	0.23	0.35	0.29
*P* value	<.001	—	<.001	<.001	<.001	<.001	<.001	<.001	<.001
SPP at T3									
*r*	0.52	0.55	1	–0.23	–0.24	–0.28	0.22	0.27	0.32
*P* value	<.001	<.001	—	<.001	<.001	<.001	<.001	<.001	<.001
Resilience at T1									
*r*	–0.28	–0.21	–0.23	1	0.45	0.39	–0.13	–0.12	–0.11
*P* value	<.001	<.001	<.001	—	<.001	<.001	<.001	<.001	<.001
Resilience at T2									
*r*	–0.24	–0.24	–0.24	0.45	1	0.48	–0.12	–0.12	–0.13
*P* value	<.001	<.001	<.001	<.001	—	<.001	<.001	<.001	<.001
Resilience at T3									
*r*	–0.17	–0.20	–0.28	0.39	0.48	1	–0.13	–0.14	–0.13
*P* value	<.001	<.001	<.001	<.001	<.001	—	<.001	<.001	<.001
IGD at T1									
*r*	0.30	0.23	0.22	–0.13	–0.12	–0.13	1	0.50	0.44
*P* value	<.001	<.001	<.001	<.001	<.001	<.001	—	<.001	<.001
IGD at T2									
*r*	0.27	0.35	0.27	–0.12	–0.12	–0.14	0.50	1	0.55
*P* value	<.001	<.001	<.001	<.001	<.001	<.001	<.001	—	<.001
IGD at T3									
*r*	0.24	0.29	0.32	–0.11	–0.13	–0.13	0.44	0.55	1
*P* value	<.001	<.001	<.001	<.001	<.001	<.001	<.001	<.001	—

a Not applicable.

### Longitudinal Invariance Test

Table 3 presents the results of the longitudinal measurement invariance tests for SPP, resilience, and IGD. The configural invariance models demonstrated satisfactory model fit across all 3 constructs (CFI and TLI values >0.90; RMSEA and SRMR values <0.08), indicating that the underlying factor structures of SPP, resilience, and IGD remained stable across the 3 measurement occasions. Metric invariance was further supported, as evidenced by minimal changes in model fit indices (ΔCFI≤0.01, ΔRMSEA≤0.015, and ΔSRMR≤0.01), confirming the stability of factor loadings and ensuring the comparability of the relationships between each latent construct and its indicators over time. Furthermore, scalar invariance was achieved, with similarly negligible changes in fit indices across waves, demonstrating the invariance of item intercepts across time points. These findings suggest that the longitudinal variations observed in SPP, resilience, and IGD reflect genuine temporal or developmental changes rather than artifacts arising from measurement inconsistency.

**Table 3.** Longitudinal measurement invariance tests for socially prescribed perfectionism (SPP), resilience, and internet gaming disorder (IGD) across 3 waves of the study among Chinese adolescents.

Model and variables	CFI^a^	TLI^b^	RMSEA^c^	SRMR^d^	ΔCFI	ΔRMSEA	ΔSRMR
SPP							
Configural invariance	0.933	0.925	0.045	0.031	—^e^	—	—
Metric invariance	0.928	0.923	0.044	0.029	0.005	0.001	0.002
Scalar invariance	0.926	0.921	0.046	0.031	0.002	0.002	0.002
Resilience							
Configural invariance	0.954	0.945	0.025	0.029	—	—	—
Metric invariance	0.953	0.943	0.024	0.023	0.001	0.001	0.006
Scalar invariance	0.953	0.944	0.023	0.021	0.000	0.001	0.002
IGD							
Configural invariance	0.943	0.912	0.048	0.047	—	—	—
Metric invariance	0.942	0.908	0.041	0.046	0.001	0.007	0.007
Scalar invariance	0.943	0.910	0.041	0.048	0.001	0.002	0.000

a CFI: comparative fit index.

b TLI: Tucker-Lewis index.

c RMSEA: root mean square error of approximation.

d SRMR: standardized root mean square residual.

e Not applicable.

### CLPM Results

**Figure 2.**
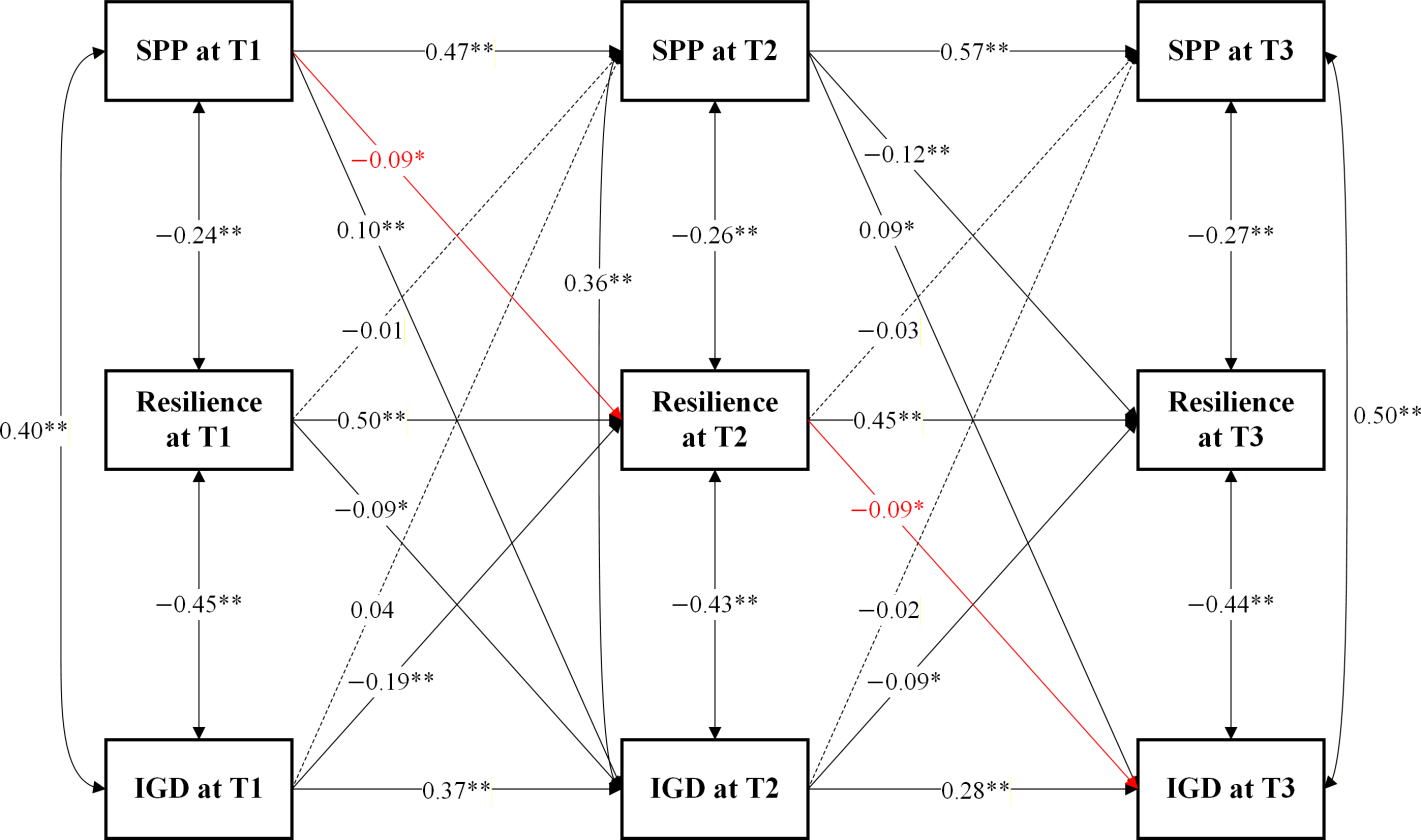
Cross-lagged panel model of socially prescribed perfectionism (SPP), resilience, and internet gaming disorder (IGD) across 3 waves of the longitudinal study among Chinese adolescents. Red arrows indicate the significant longitudinal mediation pathway from SPP at T1 to IGD at T3 through resilience at T2. *P<.01; **P<.001.

The CLPM demonstrated a good model fit to the data (*χ*^2^_38_=163.34; RMSEA=0.054; CFI=0.945; TLI=0.932; SRMR=0.047). The final interpreted model was the originally specified model, and no post hoc model respecification was undertaken. As illustrated in Figure 2, SPP at T1 significantly and positively predicted IGD at T2 (*β*=0.10, SE=0.03, 95% CI 0.04 to 0.16, *P*<.001), and SPP at T2 significantly and positively predicted IGD at T3 (*β*=0.09, SE=0.03, 95% CI 0.03 to 0.15, *P*=.004). In contrast, IGD did not significantly predict subsequent SPP across waves. With respect to resilience, SPP at T1 significantly and negatively predicted resilience at T2 (*β*=−0.09, SE=0.04, 95% CI –0.16 to –0.02, *P*=.007), and SPP at T2 significantly and negatively predicted resilience at T3 (*β*=−0.12, SE=0.03, 95% CI –0.18 to –0.06, *P*<.001). However, resilience did not significantly predict subsequent SPP. Resilience at T1 significantly and negatively predicted IGD at T2 (*β*=−0.09, SE=0.03, 95% CI –0.15 to –0.03, *P*=.001), and resilience at T2 significantly and negatively predicted IGD at T3 (*β*=−0.09, SE=0.03, 95% CI –0.15 to –0.03, *P*=.001). Meanwhile, IGD at T1 significantly and negatively predicted resilience at T2 (*β*=−0.19, SE=0.04, 95% CI –0.27 to –0.11, *P*<.001), and IGD at T2 significantly and negatively predicted resilience at T3 (*β*=−0.09, SE=0.03, 95% CI –0.15 to –0.03, *P*=.003).

Regarding longitudinal mediation, SPP at T1 significantly predicted resilience at T2, which in turn significantly predicted IGD at T3, indicating a significant indirect effect of SPP on IGD via resilience over time. Specifically, the indirect effect of SPP at T1 on IGD at T3 through resilience at T2 was significant (*β*=0.008, SE=0.002, 95% CI 0.004 to 0.012, *P*=.005).

### No Significant Sex Differences

A multigroup comparison model was used to examine sex differences in the associations among SPP, resilience, and IGD. Initially, an unconstrained model was estimated, allowing all paths to vary freely between male and female groups. The model fit was *χ*²_30_=175.80, RMSEA=0.099, SRMR=0.039, CFI=0.957, and TLI=0.906. Subsequently, a constrained model was specified, in which the corresponding paths were set to be equal across both groups. The fit indices for the constrained model were *χ*²_36_=190.39, RMSEA=0.093, SRMR=0.039, CFI=0.955, and TLI=0.917.

Model comparisons revealed that constraining the paths to be equal across sex did not lead to a substantively worse model fit, as the changes in CFI (ΔCFI=0.002) and RMSEA (ΔRMSEA=0.006) were both below the commonly recommended cutoff values (ΔCFI<0.01; ΔRMSEA<0.015). These results indicate that the longitudinal associations among SPP, resilience, and IGD did not differ significantly between males and females.

### Sensitivity Analysis

To examine whether the main findings were sensitive to the handling of missing data, multiple imputation was conducted as a robustness check. Missing data were imputed using multiple imputation with chained equations, generating 50 imputed datasets. Parameter estimates pooled across the imputed datasets were highly similar to those obtained from the primary FIML analyses. This close correspondence indicates that the main findings were robust to the method used to handle missing data and that attrition was unlikely to have materially affected the study conclusions. Detailed results of the imputation analyses are presented in Multimedia Appendix 1.

## Discussion

### Principal Findings

This study examined the longitudinal associations among SPP, resilience, and IGD across 3 waves in Chinese adolescents, with particular attention to the mediating role of resilience and potential sex differences. Consistent with the study hypotheses, higher levels of SPP predicted subsequent increases in IGD over time, and resilience served as a longitudinal mediator linking SPP to later IGD. Contrary to our third hypothesis, however, the structural associations among SPP, resilience, and IGD did not differ significantly by sex. Together, these findings suggest that SPP pressure may contribute to adolescents’ problematic gaming both directly and indirectly through lower resilience.

### The Association Between SPP and IGD

This study provides longitudinal evidence regarding the role of SPP in the development of IGD. Consistent with H1, higher SPP predicted higher subsequent IGD across both time intervals, suggesting that SPP may function as a relatively stable longitudinal risk factor for problematic gaming in adolescence. This pattern aligns with prior research showing that maladaptive perfectionistic tendencies are linked to an elevated risk of problematic internet-related behaviors and behavioral addictions among adolescents and young adults [59-61]. These findings suggest that externally imposed perfectionistic expectations may increase adolescents’ vulnerability to IGD by fostering chronic evaluative stress and making self-worth more contingent on external approval [60,61]. For adolescents with high levels of SPP, online gaming may become especially appealing because it provides a relatively controllable environment in which achievement, feedback, and social recognition can be obtained more immediately and, at least temporarily, with less fear of direct real-world evaluation [62,63]. In this way, gaming may serve not only as an escape from pressure but also as a compensatory context for restoring threatened competence and self-worth [64].

The predictive role of SPP in increasing IGD risk also highlights the importance of supportive offline environments [65,66]. Constructive academic and familial feedback may alleviate excessive self-criticism and reduce the tendency to seek external validation through online gaming platforms [65]. This implication is especially salient in the contemporary digital era, where online gaming is highly accessible and deeply integrated into adolescents’ daily routines [67]. Accordingly, interventions aimed at modifying maladaptive perfectionistic beliefs and strengthening adaptive coping strategies represent promising avenues for prevention and early intervention in problematic gaming behaviors [68,69]. Notably, this study focused primarily on a socially driven cognitive-interpersonal vulnerability (ie, SPP); future research may incorporate additional contextual and interpersonal variables, such as peer norms, academic stress, and parenting styles, to further indicate the multifaceted pathways contributing to IGD [66,70].

### The Mediating Role of Resilience

In support of H2, this study identified a significant mediating role of resilience in the longitudinal relationship between SPP and IGD. More importantly, this finding helps clarify how a socially driven perfectionistic vulnerability may be translated into problematic gaming over time [10,71]. The result is broadly consistent with the compensatory internet use framework [71,72], which proposes that maladaptive online behavior is more likely when individuals experience offline stress but lack sufficient psychological resources to cope adaptively with that stress [73]. Within this framework, resilience is not merely a general positive trait; rather, it functions as a regulatory resource that may determine whether perfectionism-related distress is managed through adaptive coping or displaced into compensatory gaming behavior [31]. Adolescents with high levels of SPP are likely to experience chronic evaluative stress, fear of negative judgment, and heightened dependence on external approval [10]. When resilience is weakened under such conditions, they may become less able to tolerate frustration, recover from perceived failure, and regulate negative affect in offline contexts [74]. Online gaming may then become especially attractive because it offers immediate feedback, structured goals, a temporary distraction, and a controllable sense of competence [75]. In this sense, our findings extend the theoretical framework by suggesting that the pathway from perfectionistic vulnerability to IGD may operate partly through erosion of a key psychological coping resource rather than through distress alone [30,72].

At the same time, our findings suggest that the role of resilience is not purely unidirectional. In addition to the hypothesized pathway from lower resilience to later IGD, IGD also negatively predicted subsequent resilience. This reverse path is theoretically meaningful because it indicates that problematic gaming may not only reflect preexisting coping difficulties but also further deplete adolescents’ psychological resources over time [76,77]. Excessive gaming may displace restorative offline experiences, weaken real-world problem-solving, disrupt sleep and daily routines, and reduce opportunities for supportive social interaction, all of which may undermine the capacity to recover from stress adaptively [78,79]. Thus, the findings point to a potentially reciprocal process: Lower resilience may increase vulnerability to gaming as a compensatory strategy, whereas higher levels of IGD may, in turn, erode resilience, thereby contributing to a self-reinforcing cycle [76,77]. However, this reciprocal dynamic did not appear to extend back to SPP, as resilience did not significantly predict subsequent SPP. This asymmetry is theoretically informative. It suggests that, although resilience is malleable and closely tied to adolescents’ ongoing coping capacity [80], SPP may represent a more stable socially driven cognitive-personality vulnerability rooted in internalized external expectations and evaluative concerns [10]. In other words, problematic gaming may weaken adolescents’ coping resources, but such depletion may not be sufficient to alter their broader perfectionistic beliefs over the relatively short time frame of the study [10,76,81]. These findings refine the proposed model by suggesting that SPP may function primarily as an upstream vulnerability factor, whereas resilience and IGD may constitute a more dynamic downstream process characterized by partial reciprocity [70,76].

It is important to recognize that, although the magnitude of the longitudinal indirect effect was small, this pattern is consistent with prior methodological and developmental research indicating that prospective effects in multiwave psychological models are typically modest in size [82]. Longitudinal mediation analyses inherently involve residualization of prior levels and multiplication of coefficients, both of which tend to attenuate effect estimates even when the underlying theoretical process is reliable [83]. Moreover, the current 3-wave design, which incorporated temporal ordering and statistical control of prior levels of both the mediator and outcome variables, followed established recommendations for testing mediational mechanisms using structural equation modeling and enables stronger causal inference than cross-sectional approaches [84]. The relatively small indirect effect may also reflect the fact that SPP is a comparatively distal cognitive-personality vulnerability, whereas IGD is a downstream behavioral outcome likely shaped by multiple intervening mechanisms. In this context, it is theoretically plausible that only a limited portion of the association between SPP and IGD would be transmitted through any single mediator, including resilience. Importantly, developmental research has emphasized that even modest cross-lagged or indirect effects may accumulate over time and retain theoretical and practical significance, particularly in adolescent psychosocial processes characterized by substantial temporal stability [85]. Accordingly, the mediation effect found in this study should not be overstated, but neither should it be dismissed as trivial. Rather, it suggests that resilience represents one meaningful, but not exhaustive, pathway through which SPP may contribute to later IGD.

Notably, resilience is unlikely to be the sole mediating pathway linking SPP and IGD. Other psychological processes, such as emotion regulation difficulties and depressive symptoms, may also play important intermediary roles [86]. For example, individuals with high levels of SPP may experience heightened emotional distress or impaired self-regulation, which may prompt them to seek immediate feedback and perceived controllability through immersive gaming environments [2,37]. The compensatory internet use framework further suggests that personality-related vulnerabilities and psychosocial resources interact dynamically in shaping excessive gaming behaviors, underscoring the value of incorporating multiple mediating variables for a more comprehensive understanding [18]. Future research should therefore examine additional mediators, such as self-esteem, coping styles, and broader personality traits, using longitudinal designs to clarify how these factors jointly influence the developmental trajectory of problematic gaming behaviors [87].

### No Significant Sex Differences in the Longitudinal Associations

Contrary to H3, the results revealed no significant sex differences in the longitudinal pathways linking SPP, resilience, and IGD. This finding suggests that the temporal associations among socially driven cognitive interpersonal vulnerability, psychological resources, and problematic gaming behaviors are largely comparable for male and female adolescents [88,89]. Importantly, although prior studies have reported sex differences in the prevalence or mean levels of IGD [88], such mean-level disparities do not necessarily indicate differences in structural or longitudinal relationships among psychological constructs. Consistent with this distinction, our results demonstrate that the cross-wave relationships among SPP, resilience, and IGD operate similarly across sex groups [90]. One plausible explanation is that the core psychological processes underlying externally imposed perfectionistic expectations and maladaptive coping, including heightened self-criticism, increased stress sensitivity, and reduced coping flexibility, may function in comparable ways for both male and female adolescents [89,91]. In addition, adolescents often encounter shared developmental challenges and environmental pressures, such as academic competition, social evaluation, and uncertainty about the future, which may contribute to relatively uniform patterns of psychological vulnerability and behavioral responses regardless of sex [92,93]. Accordingly, sex may be less influential in differentiating longitudinal psychological mechanisms than in shaping overall levels of gaming involvement or emotional symptoms [88,93]. Future research should therefore consider whether other individual or contextual factors, such as age, gaming motivation, or peer norms, explain greater variation in these longitudinal associations than sex alone.

### Implications

This study has several important implications. First, it advances the theoretical understanding of socially driven cognitive interpersonal vulnerability and behavioral addiction by demonstrating the longitudinal role of SPP in predicting IGD. Although previous studies have reported associations between perfectionism and problematic internet or gaming behaviors [23], our findings provide temporal evidence that SPP prospectively predicts IGD over time. Second, this study contributes to theoretical development in IGD research by incorporating resilience frameworks into the examination of SPP and IGD. The results indicate that adolescents with elevated SPP may experience reduced psychological resilience, which subsequently increases their susceptibility to problematic gaming behaviors. This mediating pattern supports compensatory theoretical perspectives within the behavioral addiction literature. In addition, the findings extend prior work by showing that SPP not only directly predicts IGD but also indirectly influences IGD through resilience, highlighting the importance of psychological resources as a key intermediary mechanism in the developmental pathway of problematic gaming.

From a practical perspective, these findings suggest that interventions addressing socially driven cognitive interpersonal vulnerabilities and resilience enhancement may serve as effective strategies for preventing and reducing IGD among adolescents. Because elevated SPP can weaken resilience and increase gaming risk, intervention efforts should focus on reshaping maladaptive perfectionistic beliefs, promoting flexible self-evaluation, and reducing excessive self-criticism [23]. Counseling and cognitive behavioral programs that encourage self-compassion, realistic performance standards, and adaptive responses to external expectations may alleviate the psychological pressure that fuels excessive gaming behaviors [94,95]. Furthermore, the results emphasize the value of resilience-focused initiatives in mitigating IGD risk. Schools, families, and mental health professionals may consider implementing programs that strengthen emotional regulation, coping flexibility, and problem-solving skills among adolescents [96]. Providing offline opportunities for meaningful achievement and social engagement, including group activities, mentorship programs, and counseling services, can further reinforce psychological resources and reduce reliance on online gaming as a compensatory outlet [97]. Finally, the findings highlight the importance of promoting digital self-regulation and healthy gaming education. Educational initiatives that cultivate balanced gaming habits, effective time management, and awareness of compulsive gaming tendencies may help adolescents establish a more sustainable relationship with digital entertainment and decrease the likelihood of problematic gaming involvement [98,99].

### Limitations

This study has several limitations. First, all variables were assessed using self-report measures, which may introduce social desirability and recall biases. Future research would benefit from incorporating multi-informant or behavioral assessments, such as parent or teacher reports and objective usage records. Second, although a 3-wave longitudinal design was used, this study relied on a traditional CLPM, which has important limitations. In particular, CLPM may confound stable between-person differences with within-person change processes over time [100]. Therefore, the observed cross-lagged associations should not be interpreted as definitive evidence of causal or developmental effects. Future research could use more advanced longitudinal models, such as random-intercept CLPMs, as well as experimental or intervention-based designs to provide a clearer test of within-person processes and causal relations. Third, IGD was measured using a questionnaire rather than clinical diagnostic interviews, which may limit the clinical interpretability of the findings. Replication in clinical or high-risk populations would strengthen external validity and enhance clinical relevance. Fourth, this study focused only on SPP and did not include other dimensions of perfectionism. Future longitudinal studies should examine multiple perfectionism dimensions simultaneously to compare their unique roles in the development of IGD. Finally, participants were recruited through nonrandom sampling within a limited geographic region, which may introduce sampling bias and restrict generalizability. Future research should therefore use broader and more diverse samples across cultural and socioeconomic contexts to verify the robustness of the observed associations.

### Conclusions

This study extends existing research by using a 3-wave longitudinal design to examine how SPP, resilience, and IGD are associated over time in adolescents. Unlike prior studies that have relied primarily on cross-sectional data, these findings highlight resilience as one temporal pathway through which SPP may be associated with later IGD. These results contribute to the literature by refining understanding of how socially driven perfectionistic vulnerability and psychological resources jointly shape adolescent problematic gaming. In practical terms, the findings suggest that adolescents exposed to high external evaluative pressure may benefit from early identification and prevention efforts that target both maladaptive perfectionistic beliefs and resilience-related coping capacities.

## Supplementary material

10.2196/93412 Multimedia Appendix 1 Cross-lagged panel model of socially prescribed perfectionism (SPP), resilience, and internet gaming disorder (IGD) across the 3 waves, with missing data handled using multiple imputation.

## Abbreviations

CFI: comparative fit index
CLPM: cross-lagged panel model
*DSM-5*: *Diagnostic and Statistical Manual of Mental Disorders* (Fifth Edition)
FIML: full information maximum likelihood
H1: hypothesis 1
H2: hypothesis 2
H3: hypothesis 3
IGD: internet gaming disorder
RMSEA: root mean square error of approximation
SEM: structural equation modeling
SPP: socially prescribed perfectionism
SRMR: standardized root mean square residual
TLI: Tucker-Lewis index
