# Supplementary material for: Socially Prescribed Perfectionism, Resilience, and Internet Gaming Disorder in Adolescents: 3-Wave Longitudinal Study
Source: JMIR Serious Games. 2026 Apr 30;14:e93412. doi: 10.2196/93412 (PMC13135911; doi:10.2196/93412)
Supplement: Multimedia Appendix 1 [file games-v14-e93412-s001.docx]

Figure S1 Cross-lagged panel model of SPP, resilience, and IGD across 3 waves of the longitudinal study among Chinese adolescents. Missing data were handled using multiple imputation. SPP = socially prescribed perfectionism, IGD = Internet gaming disorder. ** *p* < .01, *** *p* < .001.
